# Virtual reality in treatment of psychological disorders: a systematic review

**DOI:** 10.3389/fdgth.2026.1736381

**Published:** 2026-03-12

**Authors:** Jihane Ghorayeb, Rupa Kalahasthi, Niki Hosseini-Kamkar

**Affiliations:** Department of Liberal Arts, Rochester Institute of Technology—Dubai, Dubai, United Arab Emirates

**Keywords:** mental health illnesses, psychological disorder, randomized control trials, virtual reality therapy, systematic review

## Abstract

**Objective:**

The paper aims to systematically review the literature on the efficacy of virtual reality (VR) based therapies to treat mental health disorders in Randomized Control Trials (RCTs).

**Methods:**

As of January 2,025, three databases were searched using relevant key terms (PsycINFO, PubMed, and Medline) and Rayyan tool. Eligible studies were English-language RCTs of VR-based interventions with a clearly specified therapeutic framework (e.g., CBT) in adult psychiatric populations. We excluded pediatric or neurodevelopmental samples, animal studies, psychiatric symptoms secondary to medical conditions and those using non-immersive VR (e.g., AR) or inadequately described interventions. The three authors screened the abstracts and rotated the full review of selected papers. From the 1611 papers generated from the search results 41 were included in the systematic review, encompassing anxiety, mood, trauma-related, obsessive-compulsive, eating, substance use, and psychotic disorders.

**Results:**

Narrative syntheses of RCTs studies showed that across diagnostic categories, VR-based therapies outperformed waitlist and other inactive controls. For specific phobias, panic disorder with agoraphobia, and social anxiety, VR exposure was generally as effective as *in-vivo* exposure. For post-traumatic stress disorder, VR served as an effective alternative to imaginal prolonged exposure but did not reliably exceed it. For obsessive-compulsive disorder, findings were mixed and largely non-inferior to other treatments; for eating disorders, small trials reported comparable efficacy with occasional advantages in body image or cue reactivity. Evidence for depression, substance use, and psychotic disorders remains preliminary.

**Conclusion:**

Overall, results support VR as an effective therapeutic modality, though heterogeneity across findings limits conclusions about its superiority over established evidence-based *in-vivo* treatments. The lack of consistency across VR software and hardware, number of sessions, session duration, combination of VR with other modalities and the content of VR used make it difficult to draw comparisons across studies and generalize the findings regarding the efficacy of VR.

## Introduction

Over one billion people worldwide live with a mental illness, according to the World Health Organization's (1) report. Mental illness is defined as mental, behavioral or emotional disorders with a severity of functional impairment (2). The WHO report surveyed 144 countries revealing a critical shortage of specialized care, with two-thirds of nations reporting as few as one psychiatrist for every 200,000 people. In response, the WHO has urged not only for the training of more providers but also for the strategic adoption of digital technologies to bridge the gap between the need for and access to mental healthcare. Virtual Reality (VR) is one of these innovative tools that has gained momentum in the past decades.

In essence, VR is the use of tracking, display or other technology to immerse the user in a virtual environment (VE) which is an artificial 3-dimensional (3D) world (3). In practice VR, VE, and Immersive Reality (IR) are often used interchangeably. Various types of VR tools exist ranging from world-fixed, user-fixed to non-immersive screen displays, projectors and phones. A world-fixed example would be a cave automatic virtual environment (CAVE), and a user-fixed example would be a head-mounted display (HMDs) (4).

The integration and controlled assessment of VR technology into the field of mental health have progressed steadily since the 1990s (5, 6). Some of the clinical studies centered around exposure therapy for acrophobia (5, 6), fear of flying (7), and claustrophobia (8). Others focused on the development of assessments for treating fear of public speaking (7), assessment of body image (9), or even management of autistic spectrum disorder (10).

VR led therapies offer a safer and more controlled therapeutic environment, with adjustable stimulus intensity (11). The advantages of VR are seen in the form of client confidentiality, safety, customization to individual needs, affordability, and offering a novel approach to mental health treatment (11). For most mental health disorders, there are evidence-based interventions outlined, for example, Cognitive Behavioral Therapy for depression (12) and Exposure Therapy for anxiety disorders (13). However, a close examination of the efficacy of VR tools compared to established therapeutic modalities is needed to determine whether VR can address the global gap in mental health care, including both the shortage of accessible and affordable services and the need for treatments that are as efficacious as existing options.

Early meta-analytic evidence indicated that virtual reality exposure therapy (VRET) showed promise in reducing anxiety symptoms, although methodological limitations restricted the conclusions that could be drawn. Parsons and Rizzo (14) reviewed 21 articles and reported large effect sizes across anxiety disorders. However, some studies did not include a control group and instead relied on within-participant designs, most samples had fewer than 20 participants, some trials included non-clinical populations, and outcome measures were not uniformly standardized. Due to these limitations, the authors concluded that although VRET demonstrated large effects, inferences regarding clinical implications were difficult to establish.

More stringent evidence came from a meta-analysis that focused exclusively on anxiety disorders and between-group comparisons. Powers and Emmelkamp (15) examined 13 studies of anxiety disorders including fear of flying, acrophobia, and social anxiety. Their rigorous inclusion of randomized controlled trials with VRET as a stand-alone comparison showed that VRET was significantly more effective than control conditions and also produced improvements relative to *in vivo* exposure. Although these findings provided stronger support for VRET, the predominance of studies targeting specific phobias limited the ability to generalize to other anxiety disorders.

As the evidence base expanded, broader reviews continued to support VRET's therapeutic value while delineating its strengths and limitations. Eichenberg and Wolters (16) conducted a systematic review that included 44 studies, comprising open trials, case studies, within-participant designs, and 22 randomized controlled trials. The strongest treatment effects were observed for aviophobia, whereas panic disorder, obsessive–compulsive disorder, and post-traumatic stress disorder showed more modest results and highlighted the need for additional evidence and clearer treatment protocols. Complementing these observations, Cardoş et al. (17), reported that VRET was superior to face-to-face treatment and to control conditions in the treatment of flight anxiety at both posttest and follow-up. The magnitude of the effects was moderated by methodological factors, with stronger outcomes in smaller trials and in studies with shorter follow-up intervals.

Later systematic reviews reinforced the efficacy of VRET across a wider range of conditions while also highlighting areas requiring further research. Valmaggia et al. (18), reviewed 24 randomized controlled trials published on or after 2012 until 2015 and concluded that VR interventions were effective for phobias and promising for post-traumatic stress disorder. Their review also extended to eating disorders, autism, and schizophrenia. However, single-session interventions showed limited efficacy, and more than half of the included studies had fewer than 40 participants, with high dropout rates further reducing statistical power. Furthermore, evidence for eating disorders was particularly sparse. Building on this, Eshuis et al. (19), found that VRET was as effective as established treatments for post-traumatic stress disorder and superior to control conditions. Findings for depression have been less consistent. Evidence summarized by Fodor et al. (20), indicated that VR-based interventions may be comparable in efficacy to other active treatments. However, the limited number of randomized controlled trials in this area restricts confidence in these results and prevents firm conclusions regarding the magnitude of the treatment effect.

The most recent wave of systematic searches reflects the continued expansion of this literature and points to more nuanced results. Carl et al. (21), synthesized 30 controlled trials using random or matched assignment and found large effect sizes for VRET compared to control conditions but no significant differences when compared to *in vivo* exposure. In contrast, Wechsler et al. (22), reported a small effect favoring *in vivo* treatment for social phobia, while no significant differences emerged between VRET and *in vivo* exposure for specific phobia or agoraphobia. The most recent reviews (23, 24), that focused on anxiety disorders including social anxiety, and panic disorder with and without agoraphobia concluded that VRET was significantly more effective than control conditions but not superior to other active interventions. Other recent findings (25–27) have found VRET to be a promising mode of treatment, however, they do state that treatment integrating VR requires further research and standardization. Together, these findings suggest that while the evidence base for VRET has grown substantially, its comparative advantage over established treatments remains mixed, context dependent, and requiring further scientific research.

The standardization of technological interventions in psychopathology remains limited Cassani et al. (28),. As new software and hardware continue to evolve (29), it is increasingly important to evaluate both the efficacy and feasibility of applying VR in mental health treatment. Several gaps in the existing literature have been noted. First, most systematic reviews have focused narrowly on anxiety-related disorders, leaving limited evidence for the application of VR across other psychiatric conditions. Second, there has been inconsistent use of standardized diagnostic criteria for participant selection, with many studies failing to apply established systems such as the Diagnostic and Statistical Manual of Mental Disorders (30) or the International Classification of Diseases (31), or to use validated diagnostic tools. Third, reviews have varied widely in their methodological scope, with some including case studies and others restricting analyses to randomized controlled trials. Finally, there has been a lack of consistency in how VR is conceptualized, with some studies treating it as a tool, others as a stand-alone intervention, and still others as part of integrative evidence-based modalities.

To address these limitations, the present systematic review aims to examine the efficacy of VR within an evidence-based therapeutic framework, applied across all mental health disorders diagnosed using standardized criteria, and restricted to randomized controlled trials. In doing so, this review provides a more rigorous and comprehensive evaluation of the evidence base than prior reviews.

## Methods

The authors ran a thorough search in January 2025 of three databases (PsycINFO, PubMed, and Medline) using Rayyan and entered the following key terms “ Virtual realit* OR virtual environment* OR immersive environment* OR immersive virtual realit* AND psychiatric OR psychologic* OR pyschopatholog* OR psychophysiology OR Mood* OR PTSD OR post-traumatic stress disorder OR posttraumatic stress disorder OR dissociative subtype of PTSD OR trauma OR depression OR depress* OR anxiety OR OCD OR obsessive compulsive OR obsessive-compulsive OR schizo* OR psychosis OR psychotic OR psychosomatic OR somatoform OR conversion disorder OR neurosis OR neurotic* OR bipolar OR BPD OR manic OR personality-disorder* OR phobi* OR panic OR anorexi* OR eating-disorder* OR eating disorder* OR bulimi* ADHD OR attention deficit hyperactivity disorder OR substance use disorder* OR SUD* OR addict* OR sleep-wake disorder* OR sexual disorder* or sexual dysfunction OR neurocognitive disorder*” (Preregistration: doi: 10.17605/OSF.IO/BUXED. Note the protocol has not been changed since the registration). Where possible MeSH words were used and the search was limited to human and English language. Note the protocol has not been changed since the registration.

The inclusion criteria required that studies involve adult human participants and use a virtual reality paradigm grounded in cognitive behavioral therapy, mindfulness, or exposure therapy. Eligible studies also had to include a psychiatric population, with the exception of neurodevelopmental disorders, and be published as peer-reviewed randomized controlled trials.

Exclusion criteria were defined as follows: Studies were excluded if they were not randomized controlled trials, including conference abstracts, theses, reviews, meta-analyses, books, grey literature, or opinion pieces. Studies involving participants with childhood ADHD, autism, or other neurodevelopmental disorders were excluded, as were those involving participants younger than 18 years of age or animal studies. Studies of individuals with mental health disorders that were secondary to another biological condition, such as cancer, pregnancy, or dementia, were also excluded. In addition, articles not published, and not in English as well as studies with an unspecified or unclear VR paradigm, such as those not explicitly based on cognitive behavioral therapy, mindfulness, or exposure therapy, were excluded.

**Figure 1:**
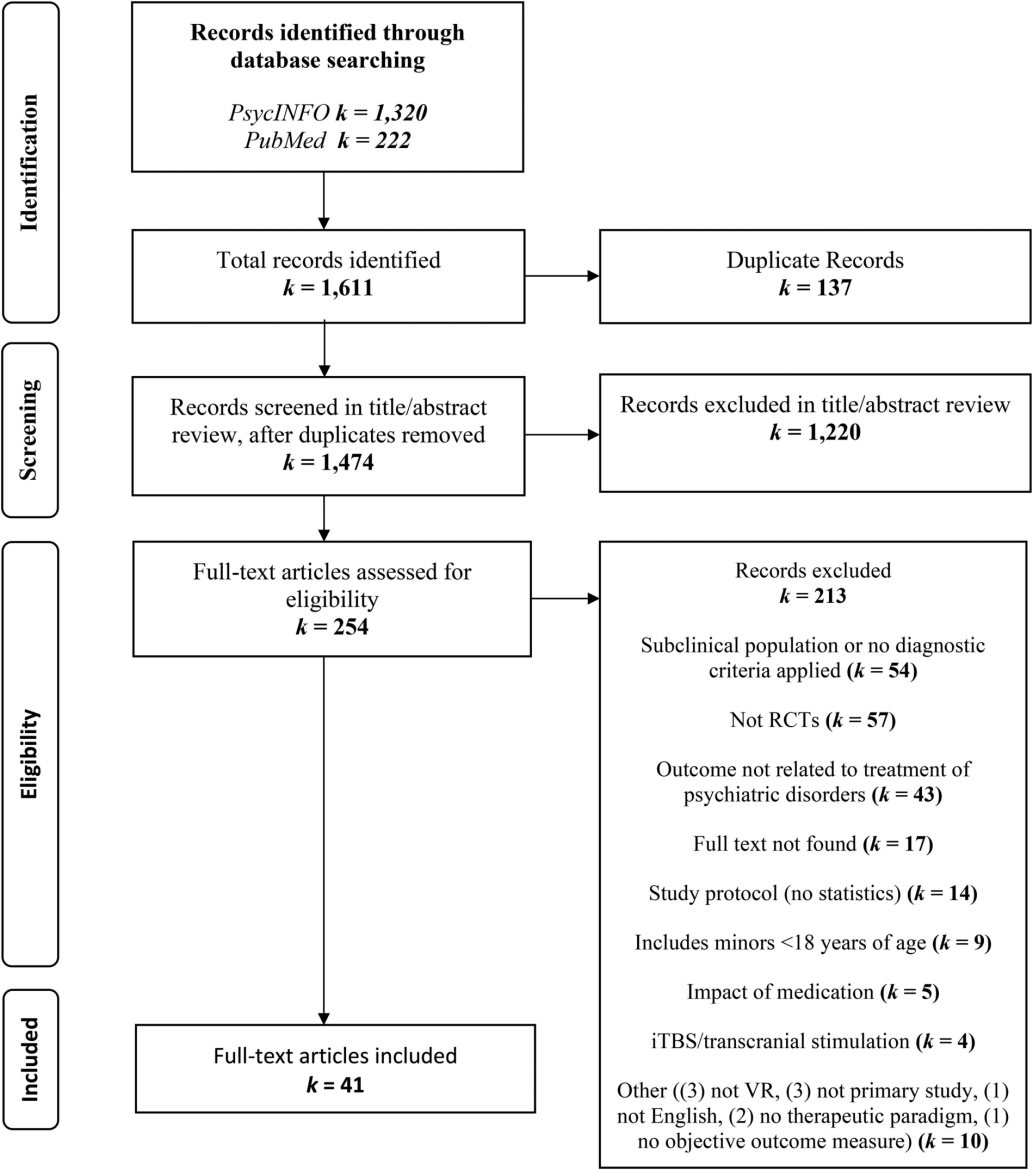
PRISMA flowchart of search yield.

A total of 1611 articles were detected across the three databases of which 137 duplicates were deleted (see PRISMA Figure 1). Two authors RK and NK screened independently for titles and abstract that fit the inclusion exclusion criteria with an inter-rater reliability of 85%. The third author JG resolved the disagreement which led to 254 eligible articles for full-text assessment. To increase thoroughness and impartial selection, full-text screenings were divided equally among pairs of authors, who rated the inclusion independently, and any disagreements were resolved through discussion with a third author until consensus was reached. Inter-rater reliability for this second step varied between: 70% and 86%. From the 254 articles screened, 41 were included in the systematic review. The primary reasons for exclusion, in descending order of frequency, were that the studies did not meet the criteria for randomized controlled trials, involved subclinical populations or diagnoses not based on the Diagnostic and Statistical Manual of Mental Disorders (DSM) or the International Classification of Diseases (ICD), or assessed virtual reality features rather than mental health outcomes.

## Results

### Heterogeneity of included studies and rationale for narrative synthesis

The systematic review yielded 41 randomized controlled trials (RCTs) meeting the inclusion criteria (see Table 1). All three authors extracted the data first independently then collaboratively. Across these studies, there was marked heterogeneity in methodological design, participant characteristics, intervention protocols, and outcome assessment, precluding the calculation of pooled effect sizes. The primary outcomes varied widely, encompassing standardized symptom severity scales (e.g., PTSD Checklist, Beck Depression Inventory, Yale–Brown Obsessive Compulsive Scale), behavioral performance measures such as behavioral avoidance tests, and physiological indices including heart rate variability and skin conductance responses. Intervention duration ranged from single-session VR exposures to structured multi-week programs extending up to 16 weeks, with the number of VR sessions varying from one to more than 20 (see Table 2). The type of VR administration also differed substantially from fully immersive virtual environments using head-mounted displays to semi-immersive or non-immersive platforms, including desktop-based VR and augmented reality systems. Moreover, the therapeutic focus ranged from exposure-based interventions targeting trauma-related cues, phobic stimuli, or obsessive–compulsive triggers, to VR-facilitated cognitive restructuring, mindfulness, or relaxation training. Control conditions were equally diverse, including waitlist and treatment-as-usual, standard face-to-face cognitive-behavioral therapy or exposure therapy, active psychotherapy, and in some cases medication-only control arms. Patient populations spanned a range of psychiatric disorders, from posttraumatic stress disorder (PTSD) and specific phobias to obsessive–compulsive disorder (OCD), schizophrenia, bipolar disorder, major depressive disorder, eating disorders, and substance use disorders, with further variability in diagnostic criteria (DSM vs. ICD) and illness severity. These methodological and clinical differences with diversity of outcome measures and control conditions, render quantitative synthesis inappropriate. Consequently, through a narrative synthesis the 41 studies were first organized by primary diagnostic category (e.g., anxiety disorders, mood disorders, trauma-related disorders, obsessive-compulsive disorder, eating disorders, substance use disorders, and psychotic disorders). This diagnostic grouping served as the primary framework for structuring the Results section.

**Table 1:** Synthesis of included articles

Study (First Author, Year)	Diagnoses	Comparisons	Key results
Anderson et al. (42)	Social anxiety disorder	VR exposure vs. group CBT vs. waitlist (WL)	VR = in vivo group; both >WL (no efficacy difference between VR and traditional exposure)
Baños et al. (72)	Stress-related disorders (PTSD/Grief/Adjustment)	VR-assisted CBT vs. standard CBT (no VR)	VR = standard CBT (both yielded similar anxiety reduction)
Bouchard et al. (43)	Social anxiety disorder	VRCBT vs. CBT vivo vs. waitlist control	Both CBT in vivo and CBT in virtuo showed significant improvement over waitlist; no statistical difference between active conditions except that CBT in virtuo was more effective to reduce symptoms post and 6 months follow-up
Cesa et al. (58)	Binge Eating Disorder (with obesity)	VR-enhanced CBT vs. standard CBT vs. inpatient program	All groups improved; no significant differences between conditions in weight loss, though VR group showed better body image outcomes
Difede et al. (51)	PTSD (combat-related)	VR exposure vs. imaginal exposure (both with D-cycloserine)	VR = imaginal (both groups had large PTSD symptom reductions; no advantage to VR when combined with medication)
Emmelkamp et al. (40)	Acrophobia (height phobia)	VR exposure vs. in vivo exposure	VR = in vivo (both effective in reducing fear of heights and avoidance)
Ferrer-García et al. (56)	Bulimia Nervosa/Binge ED	VR cue-exposure therapy vs. additional standard CBT sessions	VR cue exposure >CBT (VR produced greater reduction in binge frequency and craving)
Gamito et al. (73)	PTSD (elderly veterans)	VR exposure vs. imaginal exposure vs. WL	VR > WL (PTSD and anxiety symptoms dropped with VR); VR ≈ imaginal (no clear difference between active treatments in this pilot)
Garcia-Palacios et al. (37)	Spider phobia	VR exposure vs. waitlist control	VR > WL (VR group showed significant fear and avoidance reduction compared to no treatment)
Geraets et al. (62)	Psychosis (Paranoid Ideation)	VR CBT vs. treatment-as-usual (TAU)	VR CBT > TAU (greater decreases in paranoid thoughts and anxiety in daily life after VR therapy)
Javaherirenani et al. (54)	Obsessive–compulsive disorder (contamination subtype)	VR exposure + response prevention (ERP) vs. standard therapy (no VR)	VR ERP > control (VR group had larger OCD symptom reduction than those without VR exposure)
Kampmann et al. (44)	Social anxiety disorder	VR exposure vs. in vivo exposure vs. WL	VR = in vivo; both > WL (active treatments produced equivalent improvements in social anxiety)
Kim et al. (74)	Social anxiety disorder	Self-guided VR exposure vs. waitlist	VR > WL (significant decrease in anxiety and avoidance with at-home VR training compared to no treatment)
Maltby et al. (34)	Flying phobia (with some panic/agoraphobia cases)	VR exposure therapy vs. attention-placebo group therapy	VR > placebo (VR group showed significantly greater anxiety reduction and a higher rate of clinically significant improvement)
McLay et al. (75)	PTSD (combat-related)	VR exposure therapy vs. treatment-as-usual (TAU)	VR ≈ TAU (no significant difference in PTSD symptom outcomes between VR and standard care in this study)
McLay et al. (76)	PTSD (combat-related)	VR exposure (new protocol) vs. traditional exposure therapy	VR = traditional (PTSD symptoms improved similarly in both groups; no between-group difference)
Meyerbroeker et al. (49)	Agoraphobia	CBT VRET vs. CBT Exposure in vivo vs. no treatment	The two active conditions were superior to no treatment. No significance difference between the two active treatment for agoraphobia reduction
Michaliszyn et al. (38)	Spider phobia	VR in virtuo exposure vs. in vivo exposure (vs. minimal control)	VR = in vivo; both > control (virtual and real exposures were equally effective, each outperforming minimal intervention)
Miloff et al. (39)	Spider phobia	Automated VR exposure (self-guided) vs. one-session therapist-led in vivo exposure	VR ≈ in vivo (virtual therapy was non-inferior to the gold-standard one-session treatment in reducing phobic fear)
Moldovan et al. (70)	Social Anxiety	CBT VR vs. waitlist	No significant difference between the two groups on all measures with one session CBT VR
Mühlberger et al. (69)	Flying phobia	Repeated VR flight simulations: with vs. without adjunct relaxation	*(No external control group)* Both VR groups showed anxiety reduction; adding relaxation did not significantly alter outcomes (both approaches led to decreased flight fear over sessions)
Pallavicini et al. (67)	Generalized anxiety disorder	VR anxiety management program with biofeedback vs. the same VR program without biofeedback vs. WL	VR (with or without biofeedback) >WL (significant improvement in GAD symptoms in both VR groups; physiological biofeedback did not yield extra benefit over VR alone)
Castro et al. (47)	Agoraphobia (chronic)	CBT+ VR exposure vs. CBT+ in vivo exposure vs. antidepressant medication alone	CBT (with VR or in vivo) >meds-only (both exposure-based therapy groups achieved superior outcomes to medication-only on agoraphobia measures; no major difference between VR and real exposure groups)
Pitti et al. (48)	Agoraphobia	CBT+ in vivo exposure vs. CBT+ VR exposure vs. exposure therapy alone	Combined CBT+ Exposure (with or without VR) >exposure alone (adding CBT skills enhanced outcome). VR vs. in vivo combined approaches: no significant difference (both combos effective)
Pot-Kolder et al. (61)	Psychosis (Paranoid Ideation)	VR social CBT vs. waitlist control	VR > WL (significant reductions in paranoid delusions and social avoidance in the VR therapy group compared to no treatment)
Ready et al. (53)	PTSD (Vietnam veterans)	VR exposure therapy vs. present-centered therapy (PCT)	VR ≈ PCT (both groups showed some PTSD symptom improvement; no clear advantage to VR in this small sample)
Reger et al. (50)	PTSD (active-duty military)	Prolonged exposure: VR-based vs. imaginal vs. waitlist	VR = imaginal; both >WL (no significant outcome difference between VR exposure and traditional imaginal exposure)
Riva et al. (57)	Binge eating disorder and obesity	Pre and post measures of VREDIM Experiential Cognitive therapy	There is a significant reduction of body image and body satisfaction after 5 sessions of VR Experiential cognitive therapy
Riva et al. (71)	Binge eating disorder	VREDIM Experiential Cognitive Therapy vs. CBT vs. nutritional group vs. waitline	ECT treatment is able to reduce binge frequency better than CBT and nutritional groups after a 6-month follow-up. ECT was more effective than CBT in improving the overall psychological state of the patients. In particular, ECT was more successful than CBT in improving body satisfaction and resistance to social pressure.
Robillard et al. (45)	Social anxiety (public speaking)	VR CBT (with virtual audiences) vs. traditional group CBT vs. WL	VR = group CBT; both >WL (preliminary evidence that VR and face-to-face group therapy yielded comparable improvements in social anxiety relative to no treatment)
Rothbaum et al. (32)	Flying phobia	VR exposure vs. in vivo exposure vs. WL	VR = in vivo; both >WL (VR and real exposure both produced large fear reductions, outperforming waitlist
Rothbaum et al. (33)	Flying Phobia	VR exposure vs. in vivo exposure vs. WL	VR = in vivo; both >WL (consistent with earlier findings – VR worked as well as standard exposure and better than no treatment)
Rus-Calafell et al. (36)	Flying phobia	VR exposure vs. imaginal exposure (one-session each)	VR = imaginal in short-term efficacy; VR showed continued improvement after a real test flight, whereas imaginal did not (VR group-maintained gains at follow-up)
Triscari et al. (35)	Flying phobia (Flight Anxiety)	CBT+ systematic desensitization vs. CBT+ EMDR vs. CBT+ VR exposure	All groups improved significantly; no significant differences (all three combinations effectively reduced flight anxiety to a similar degree)
Van Gelderena et al. (52)	PTSD (treatment resistant veterans)	3MDR (VR) vs. non-specific treatment component control group (NTCC)	Virtual intervention led to significant improvements in PTSD symptom severity as compared to the control condition with a large effect size
Veling et al. (64)	Mixed Psychiatric (Anxiety/Depression/Psychosis)	VR relaxation (interactive VR nature scenes) vs. traditional relaxation exercise	Both methods reduced stress; VR > standard relax (VR led to a significantly greater immediate reduction in anxiety and negative affect than the audio-guided relaxation)
Vincelli et al. (46)	Panic Disorder with Agoraphobia	VR-enhanced experiential therapy vs. standard CBT vs. WL	VR = CBT; both >WL (both active treatments eliminated or sharply reduced panic attacks and outperformed waitlist on anxiety measures)
Wan et al. (65)	Chronic Insomnia	VR therapy program (6 weeks) vs. medication management control	VR > control (the VR group had a greater improvement in sleep quality and larger decreases in insomnia severity, as well as anxiety/depression symptoms, compared to control)
Zadbar et al. (55)	Obsessive–compulsive disorder	VR exposure therapy vs. CBT vs. waitlist control	VR = CBT; both >control (patients in both active treatment arms showed equivalent OCD symptom reduction, each superior to no treatment condition)
Zainal et al. (68)	Social anxiety disorder	VRE vs. waitlist(WL)	VRE vs. WL resulted in greater reductions in SAD symptom severity, job interview fear, and trait worry, with moderate-to-large effect sizes from pre-to-post treatment. The results did not reach significance.
Zhang et al. (59)	Alcohol dependence	VR cue exposure therapy vs. treatment-as-usual (TAU)	VR > TAU (VR group demonstrated larger decreases in alcohol craving and reactivity; greater physiological calm in response to alcohol cues than control group)

**Table 2:** Summary of VR protocols across studies.

	Study (First Author, Year)	Diagnoses	Number of VR sessions	Duration of VR sessions	VR protocol description
1	Anderson et al. (42)	Social anxiety disorder	8 sessions	Not specified	The VR protocol consisted of progressively challenging virtual speaking environments, including a small conference room (approximately five audience members), a classroom setting (around 35 audience members), and a large auditorium (up to 100 audience members). Therapists dynamically controlled audience behaviors, such as displaying interest, boredom, support, hostility, or distraction (e.g., phone ringing). Virtual audience members could also ask standardized or individualized questions delivered via therapist voiceover to increase realism and task difficulty.
2	Baños et al. (72)	Stress-related disorders (PTSD/Grief/Adjustment)	9 weekly sessions for all PTSD For AD, 4–6 weekly sessions	1.5–2 h	The VR intervention used *EMMAs World*, an adaptive virtual environment that allows patients to explore stress-inducing scenarios tailored to their therapeutic needs. The system incorporates customizable emotional virtual elements that can be activated to support targeted emotional processing during treatment
3	Bouchard et al. (43)	Social anxiety disorder	8 sessions in total of the 14 weeks each session lasting 60 min	20–30 min	Participants were immersed using a head-mounted display with motion tracking and exposed to eight VR-based social scenarios, including public speaking, job interviews, social interactions in private and public settings, and situations involving criticism or social pressure. A neutral environment was used initially to familiarize patients with the VR system. Scenarios were collaboratively selected by the therapist and patient based on individual therapeutic needs. Participants navigated the virtual environments while verbally interacting with virtual characters, whose responses were therapist-controlled, and could complete sessions either seated or standing depending on the scenario
4	Cesa et al. (58)	Binge eating disorder (with obesity)	15 sessions	60 min	VR sessions used the open-source NEUROVR platform, which includes 14 environments targeting relapse and maintenance-related situations, along with two body image comparison settings. The VR scenarios were designed to facilitate practice of eating regulation, emotional and interpersonal coping, decision-making, and problem-solving skills.
5	Difede et al. (51)	PTSD (combat-related)	9 sessions	90 min	In prolonged exposure sessions, participants engaged in imaginal exposure by recounting their traumatic experiences aloud in the present tense, following established exposure therapy procedures.
6	Emmelkamp et al. (40)	Acrophobia (height phobia)	3 sessions	60 min with breaks	In the initial VR session, participants were briefly familiarized with the virtual environment. They were then gradually exposed to three purpose-built VR settings: a multi-level shopping mall, a fire escape approximately 50 feet high, and a rooftop garden about 65 feet above ground with city views. Exposure was conducted hierarchically, with therapists providing ongoing verbal guidance and encouragement throughout the session
7	Ferrer-García et al. (56)	Bulimia Nervosa/Binge ED	6 sessions	60 min	Prior to treatment, participants used VR software to develop individualized exposure hierarchies based on craving and anxiety responses. Cravings were rated on a 0–100 scale for 30 virtual food cues and four binge-related environments (e.g., kitchen, dining room), which were used to generate 40 personalized 3D VR exposure scenarios. Anxiety ratings collected after brief VR exposures were then used to finalize the hierarchy.
8	Gamito et al. (73)	PTSD (elderly veterans)	12 sessions	Not specified	The VR environment was created using the Hammer graphics editor (Valve Corp.) and depicted a forested footpath scenario in which participants followed a column of virtual soldiers. The exposure included combat-related cues such as ambushes, gunfire, tracer bullets, explosion sounds, and periods of waiting for evacuation
9	Garcia-Palacios et al. (37)	Spider Phobia	Ranged 3–10 sessions	60 min	Participants underwent immersive VR exposure with tactile augmentation using a head-mounted display and position-tracking devices. A wide field-of-view VR helmet provided a stereoscopic, interactive 3D environment, while a position-tracking system monitored head and hand movements and the virtual stimulus location. Exposure took place in SpiderWorld, a modified version of KitchenWorld, designed to elicit fear responses in a controlled virtual setting.
10	Geraets et al. (62)	Psychosis (Paranoid Ideation)	16 sessions	60 min	The treatment protocol was aimed at reducing paranoia and improving social participation. The therapy consisted of evidence-based CBT elements such as making treatment goals, exposures, behavioral experiments, reducing safety behavior, and attention strategies. However, instead of in vivo, exercises and behavioral experiments were done in VR. In the first two sessions personal goals were set, and the VR system was introduced. In the following sessions, participants practiced 40 min within VR each session. No homework was given
11	Javaherirenani et al. (54)	Obsessive–compulsive disorder (contamination subtype)	12 sessions	25–45 min	Participants were immersed in a virtual house featuring graded contamination scenarios ranging from mild to severe. Using a joystick and motion tracker, they navigated the environment and reported distress levels (SUDS, 1–10) during exposure. The initial session focused on familiarization with the VR system through exploration of neutral spaces such as the kitchen and bathroom. Subsequent sessions followed a hierarchical exposure to contamination-related stimuli, progressing from minimally soiled objects to highly contaminated items (e.g., dirty bins, rotten food), with anxiety reduction as the criterion for advancement. Compulsive responses were prevented within VR (e.g., nonfunctional faucets)
12	Kampmann et al. (44)	Social anxiety disorder	7 sessions bi-weekly	30 min exposure twice; separated by 5 min breaks	Virtual exposure was delivered using the Delft Remote Virtual Reality Exposure Therapy (DRVRET) system, developed with Vizard v3.0 software. The setup comprised three interconnected workstations: one running the VR server and data logging, a second operating the VR environment with mirrored real-time display for therapist monitoring, and a third allowing the therapist to control virtual scenarios. Participants wore an nVisor SX head-mounted display providing stereoscopic projection
13	Kim et al. (74)	Social anxiety disorder	3 weeks (number not specified)	Not specified	The VR protocol involved delivering speeches in three simulated environments (school, business, daily life), each with four situations of increasing difficulty. Using a Samsung Gear VR headset, participants completed 8 sessions over two weeks at a clinic, progressing through 24 topics.
14	Maltby et al. (34)	Flying phobia (with some panic/agoraphobia cases)	5 sessions	(Session 1) 90 min, (Sessions 2–5) 50 min	The VR environment was powered by an Intergraph TDZ 2000 computer and delivered through a Virtual Research V6 head-mounted display with integrated earphones for visual and auditory immersion.
15	McLay et al. (75)	PTSD (combat-related)	10 weeks; 4–20 sessions (8.8 average)	Not specified	VR-GET integrates graded virtual reality exposure, physiological monitoring, and skills training. It uses VR simulations of Iraq or Afghanistan tailored to a participants most salient traumatic experience
16	McLay et al. (76)	PTSD (combat-related)	8–12 sessions (VR sessions not specified)	90 min	Two interchangeable virtual reality systems were employed. The first, “Virtual Iraq/Afghanistan,” developed by USC and Virtually Better, offered optional scent and vibration features. The second system, from the Virtual Reality Medical Center, similarly recreated wartime settings. Both utilized head-mounted displays with 3D graphics and audio, producing video game-like rather than photorealistic environments.
17	Meyerbroeker et al. (49)	Agoraphobia	10 sessions	Not specified	The 10-session therapy comprised two modules: CBT components and exposure therapy. The critical difference between treatment conditions was the exposure method: either *in vivo* or via VR (using an HMD or CAVE system). The VR exposure involved practicing in seven different virtual environments, such as a supermarket, metro station, and town square, where crowd density was gradually increased to grade the difficulty.
18	Michaliszyn et al. (38)	Spider phobia	8 sessions	1.5 h with breaks	The virtual environments were rendered on a PC and displayed through a monoscopic head-mounted display (HMD) with an 800 × 600 resolution, draped with a black cloth to block ambient light. Head movement was tracked using a 3-degree-of-freedom motion sensor.
19	Miloff et al. (39)	Spider phobia	1 session	3 h	VIMSE was a fully automated, 3 h VR exposure therapy application based on one-session treatment principles. Participants progressed at their own pace through eight levels of spider exposure, providing fear ratings and receiving feedback from a virtual therapist, while a human therapist provided technical and emotional support. The protocol included gamified elements such as points and puzzles.
20	Moldovan et al. (70)	Social anxiety	1 session	1.5 h	Following a CBT session where a personalized fear hierarchy was established, VR exposure was tailored to this hierarchy. For social and flight phobia, a head-mounted display (HMD) was used, while acrophobia treatment utilized a CAVE system. The single-session exposure consisted of four 15 min scenarios. Fear was monitored using Subjective Units of Discomfort (SUDs, 0–100), with ratings provided every 3 min and during challenging moments.
21	Mühlberger et al. (69)	Flying phobia	6 sessions	16 min	The VR system was developed by the Fraunhofer IGD in Germany. It provided visual, auditory, and motion stimuli using a head-mounted display (VR4 HMD). Head movement was tracked to adjust the visual field in real time, with rendering handled by a high-performance graphics computer. The environment achieved a detailed, high frame rate by forgoing stereoscopic display.
22	Pallavicini et al. (67)	Generalized anxiety disorder	8 weeks (number not specified)	Not specified	The VR system utilized a laptop, a Vuzix head-mounted display, a therapists netbook for control and monitoring, and a joystick for navigation. During the protocol, following an initial clinical evaluation and a 3 min baseline physiological recording, patients explored a virtual tropical island to perform guided relaxation exercises. For the VRMB group, elements of the environment were modified in real-time based on the patients heart rate, providing integrated biofeedback. After the session, physiological data was recorded again, a final clinical evaluation was conducted, and patients were assigned daily relaxation homework using a smartphone.
23	Castro et al. (47)	Agoraphobia (chronic)	8 sessions	30–45 min	The virtual environments were seven possible airports building and a plane, a square and a street, an elevator and an underground car park, a bank. (not described in this study)
24	Pitti et al. (48)	Agoraphobia	8 sessions	12–15 min	The exposure was conducted using a VR system controlled by an Intel computer, where participants navigated the environments with a wireless joystick and experienced immersive 3D sound through a surround audio system.
25	Pot-Kolder et al. (61)	Psychosis (Paranoid Ideation)	16 sessions	1 h 40 min	Four virtual social environments (street, bus, café, supermarket) were developed for exposure therapy. Using a gamepad and a head-mounted display, participants navigated these spaces while a therapist dynamically customized the scenarios by controlling the number, appearance, and behavior of avatars—including triggering hostile interactions or statements—to personally tailor the exposure to the individuals paranoid fears.
26	Ready et al. (53)	PTSD (Vietnam veterans)	10 sessions	90 min	The VR exposure therapy protocol for war-related trauma utilized a head-mounted display and joystick for navigation within two customizable virtual Vietnam environments: a jungle landing zone and a Huey helicopter flight. The therapist controlled all visual and auditory stimuli—such as gunfire, explosions, and helicopter sounds—to gradually present trauma-related cues tailored to the patients personal experiences. During exposure, patients were guided to mentally stay grounded in the present while revisiting traumatic memories
27	Reger et al. (50)	PTSD (active-duty military)	10 sessions	90–120 min	Virtual Reality Exposure (VRE) followed the standard Prolonged Exposure (PE) protocol, with two key modifications. First, patients revisited their traumatic memories while wearing a head-mounted display (HMD) in a tailored VR environment, describing the event in the first person, present tense with their eyes open as the therapist matched the virtual scene to their account. Second, an additional introductory session familiarized patients with the VR equipment using a calm virtual park environment
28	Riva et al. (57)	Binge Eating Disorder and Obesity	10 sessions	50 min	Therapists utilized VREDIM (Virtual Reality for Eating Disorders Modification). This system consists of 14 virtual environments
29	Riva et al. (71)	Binge Eating Disorder	5 sessions	Not specified	VEBIM 2 is implemented on a Thunder 500/C virtual reality system by Virtual Engineering of Milano, Italy. The Thunder 500/C is a Pentium III based immersive VR system (500 mHz, 128 mega RAM, graphic engine: Matrox MGA 400 Max 32MB WRAM) including a head mounted display (HMD) subsystem.
30	Robillard et al. (45)	Social Anxiety (public speaking)	16 sessions	Not specified	Not specified
31	Rothbaum et al. (32)	Flying Phobia	4 sessions	60 min	The virtual reality exposure therapy simulated the experience of flying, including sitting in an airplane, take-offs and landings, and flights in both calm and stormy weather. The patients chair was enhanced with a subwoofer to generate realistic noise and vibrations synchronized with key moments during the simulation.
32	Rothbaum et al. (33)	Flying Phobia	4 sessions	Not specified	The VR exposure setup for flight anxiety consisted of a chair fitted with an actual airline seatbelt and a bass speaker platform underneath for vibration. Participants wore an immersive headset with head tracking while a therapist controlled the session from a keyboard, advancing through a graded simulation of a commercial flight. This simulation included the full sequence from boarding and takeoff to flying in both calm and stormy weather, followed by landing
33	Rus-Calafell et al. (36)	Agoraphobia	6 sessions	60–75 min	The VR condition utilized the Virtual Flight® program, which provided customizable exposure parameters including gender, weather, time of day, and turbulence level. The experience progressed through three distinct scenarios: a preparatory room for packing and obtaining a boarding pass; an airport terminal featuring plane sightings and flight announcements; and a full flight sequence aboard a plane, complete with ambient sounds, take-off, and adjustable turbulence.
34	Triscari et al. (35)	Flying Phobia (Flight Anxiety)	3 sessions	2 h	A controlled, simulated environment where patients can confront feared objects or situations. (not specified)
35	Van Gelderena et al. (52)	PTSD (treatment resistant veterans)	6 sessions	70–90 min	The 3MDR protocol combines walking on a treadmill with exposure to a 180° virtual reality environment that displays personalized, deployment-related images and music selected by the patient. While walking in a safety harness, the patient approaches a series of images, describes associated memories, performs a tracking task, and rates their distress, with continuous therapist support.
36	Veling et al. (64)	Mixed psychiatric (Anxiety/Depression/Psychosis)	10 sessions	10 min	The VRelax system was operated using a Samsung smartphone connected to a Gear VR head-mounted display, with three-dimensional audio delivered via headphones. Participants began each session in a virtual waiting room where initial assessments were completed, before transitioning to a beach environment. From there, they could navigate to a variety of 360-degree nature scenes—such as coral reefs, mountain meadows, or drone flight landscapes—by focusing on visual hotspots. The available environments included peaceful settings like underwater dolphin encounters and a beach session featuring Tibetan singing bowl therapy.
37	Vincelli et al. (46)	Panic disorder with agoraphobia	8 sessions	Not specified	The VEPD system, which comprises four virtual environments—an elevator, a supermarket, a subway, and a large square—designed for exposure therapy. The therapist customizes each scenarios anxiety-inducing elements via a control menu.
38	Wan et al. (65)	Chronic insomnia	3 sessions + 6 sessions at home	30 min	The 6-week VR therapy protocol consisted of daily 30 min sessions divided into three sequential phases, each lasting two weeks. Each phase paired a core therapeutic exercise—beginning with breath relaxation, progressing to mindfulness mood observation, and concluding with focused breath meditation—with a corresponding serene virtual environment to support graduated skill development
39	Zadbar et al. (55)	Obsessive–compulsive disorder	12 sessions	8 min	The VR exposure protocol utilized first-person scenarios involving contamination and other anxiety-provoking situations, with four specific environments selected based on a prior pilot stud
40	Zainal et al. (68)	Social Anxiety disorder	4 sessions	50–60 min	The VR protocol utilized a Pico Goblin headset to deliver 360° stereoscopic video scenarios, integrated with biometric feedback and adaptive machine-learning prompts. Participants selected one of two social exposure themes—an informal dinner party or a formal job interview—each comprising a graded sequence of scenarios targeting specific social fears. A virtual therapist guided users through each scene, providing instructions and CBT rationale. Subjective distress (SUDS) was assessed at multiple points using head-tracking to select ratings. The system automatically adjusted prompts based on detected user engagement via voice and head tracking, with all sessions monitored wirelessly by a researcher.
41	Zhang et al. (59)	Alcohol dependence	8 sessions	8 min	The VR cue exposure scenes were captured with an Insta360Pro2 panoramic camera and processed for playback on Oculus Quest equipment. VR-based offered a significant highly realistic, multisensory environments and delivered multiple exposure cues simultaneously.

Within each diagnostic category, studies were further categorized based on the type of VR-based therapeutic approach, including VR-assisted exposure therapy, CBT-informed VR interventions, mindfulness-based VR interventions, and hybrid or multimodal VR applications. Additional study characteristics were descriptively compared, including control conditions (e.g., wait-list, treatment as usual, *in-vivo* exposure, imaginal exposure), intervention dosage (number and duration of sessions), and VR delivery features (hardware and software platforms, when reported).

Outcomes were synthesized by examining patterns of effectiveness of VR interventions relative to inactive and active control conditions within each diagnostic and modality subgroup. Emphasis was placed on identifying consistencies, non-inferiority patterns, and sources of variability across studies rather than generating pooled effect estimates.

This structured narrative approach allowed for transparent comparison across clinically diverse studies while highlighting both convergent and divergent findings across studies within each category.

### Anxiety disorders

#### Fear of flying

The included trials (*k* = 6) evaluating VRET for fear of flying consistently indicated that VR efficacy was comparable to traditional *in vivo* exposure, with some superiority over waitlist and placebo controls. In a three-arm RCT (32, 33), found that both VR exposure and *in vivo* exposure significantly reduced flight-related anxiety compared to a waitlist, with no difference between the active interventions. Maltby et al. (34), further reported that VR exposure produced greater reductions in flight anxiety than nonspecific group treatment as a placebo, with large within-group improvements from pre- to post-treatment. Comparative trials with active interventions further support these findings; for example, Triscari et al. (35), randomized participants to CBT with systematic desensitization, CBT with eye movement desensitization and reprocessing (EMDR), and CBT with VR exposure. All three approaches were equally effective in reducing flying anxiety. The results indicate that VR exposure, when integrated into CBT, were as efficacious as other established adjunctive methods. Longitudinal data (36) suggested continued improvement for the VR group following real post-treatment test flight and at follow up.

#### Spider phobias

The evidence base for VR exposure in spider and small animal phobias (k = 3) is favorable, although most trials demonstrate equivalence rather than superiority to *in vivo* exposure. For example, Garcia-Palacios et al. (37), found that VR exposure produced significantly greater reductions in fear and avoidance than a waitlist control, with large between-group effect sizes. In terms of comparison with *in vivo* exposure, VRET was found to be as effective as *in vivo* exposure (38), however, participants in the *in vivo* group demonstrated significant and continued gains after the posttest as compared to VRET. Alternative delivery modalities have expanded accessibility. In a non-inferiority RCT for spider phobia, Miloff et al. (39), found that automated VR exposure (minimal therapist involvement) was as effective as a single-session therapist-led *in vivo* exposure, with both yielding large improvements in fear.

#### Acrophobia

Like other specific phobias, the evidence base of acrophobia suggests VRET being as efficacious as *in vivo* exposure (k = 2). Emmelkamp et al. (40), found both VR and *in vivo* exposure to significantly reduced fear of heights relative to waitlist, with gains maintained at follow-up. Some studies have examined the influence of immersion on treatment outcomes. Krijn et al. (41), compared low-presence VR delivered via a head-mounted display with high-presence VR using a computer automatic virtual environment (CAVE) for acrophobia. Both VR formats were more effective than no-treatment, with no significant differences in efficacy between the two conditions., and results were maintained at six-month follow-up.

#### Social anxiety disorder

A growing body of literature (k = 6) has examined the efficacy of virtual reality VR–assisted therapy for social anxiety disorder (SAD), particularly in relation to public speaking anxiety. Across most trials, VR exposure therapy yields significant reductions in social anxiety symptoms, with outcomes broadly comparable to those achieved through traditional *in vivo* exposure therapy. With participants randomly assigned to VRET, *in vivo* exposure, or a waitlist control, Anderson et al. (42), found significantly greater improvements in social anxiety for both treatment groups compared to the waitlist, however, no significant differences between VRET and *in vivo* exposure. Similarly, in comparing CBT with VR exposure, CBT with *in vivo* exposure and waitlist control groups, Bouchard et al. (43), found that both CBT approaches led to substantial and statistically comparable reductions in social anxiety, with improvements sustained at the 6-month follow up.

However, not all VR interventions produce effects comparable to therapist-led *in vivo* exposure. Kampmann et al. (44), found that both VR exposure with verbal interaction and *in vivo* exposure improved social anxiety symptoms, speech duration, and perceived stress, and avoidant personality disorder–related beliefs compared to a waitlist, with some gains maintained at follow-up. However, *in vivo* exposure produced broader improvements, including greater reductions in fear of negative evaluation, general anxiety, and depression, as well as superior outcomes for social anxiety symptoms at post-treatment and follow-up. Similarly, Robillard et al. (45), demonstrated reductions in fear of negative evaluation and public speaking anxiety after interaction with relatively basic VR avatars, but these gains were more modest than those reported in more immersive, therapist-guided protocols.

#### Panic disorder with agoraphobia

VR exposure for panic disorder with agoraphobia consistently yields clinically meaningful symptom reduction and, in most studies, matches the efficacy of gold-standard *in vivo* methods (k = 7). It is important to note that while some studies focused primarily on panic with agoraphobia, others did not view it as a standalone diagnosis. Early controlled research by Vincelli et al. (46), found that VR-assisted CBT for panic disorder with agoraphobia achieved symptom reductions comparable to standard CBT, while requiring 33% fewer sessions, suggesting equivalent efficacy with greater efficiency.

Subsequent trials have examined VR as both a stand-alone modality and an adjunct to conventional interventions. Castro et al. (47), reported that, among patients with long-term agoraphobia already receiving antidepressant medication, adding VR exposure to CBT produced outcomes comparable to traditional CBT, with both outperforming a medication-only condition. Similarly, Pitti et al. (48), found that combining paroxetine with CBT was effective for agoraphobia, whether exposure was delivered *in vivo* or via VR. Both combined treatments outperformed medication alone, with the VR group showing somewhat greater gains in confronting phobic stimuli. Meyerbroeker et al. (49), found that both CBT plus VR exposure and CBT plus *in vivo* exposure were significantly more effective than no treatment for panic disorder with agoraphobia. However, while most outcomes were equivalent, *in vivo* exposure showed a slight advantage on panic severity, leading the authors to caution that, given implementation costs and limited long-term data, VR should be considered a viable alternative rather than a replacement for traditional exposure.

### Post-Traumatic stress disorder (PTSD)

VRET for PTSD produces clinically meaningful symptom reductions, but when compared directly with standard imaginal prolonged exposure (PE), results are mixed and show no consistent advantage for either approach (k = 8). In a large, randomized trial with active-duty soldiers, Reger et al. (50), found that both PE and VRET significantly reduced PTSD symptoms compared to a waitlist, with similar dropout rates and no overall difference in symptom reduction at post-treatment. Follow-up analyses showed slightly greater improvements for PE at three and six months. Similarly, Difede et al. (51), reported comparable symptom reductions with VRET and PE when paired with D-cycloserine (DCS) or placebo. Together, these findings suggest that VRET is an effective alternative to PE but does not consistently outperform it.

At the same time, some findings indicate that VR may have situational advantages for certain patients. In treatment-resistant combat veterans, van Gelderen et al. (52), tested a VR-based protocol (3MDR) and observed larger symptom reductions than a non-trauma-focused active control. Because the comparator differed from imaginal exposure and the sample was specialized, these results point to potential utility in hard-to-treat subgroups rather than generalized superiority. Early feasibility work in veterans also suggested that VR can facilitate engagement with trauma cues, though small samples limited statistical inference regarding efficacy (53).

### Obsessive-Compulsive disorder (OCD)

As a relatively recent area of investigation, findings of the efficacy (k = 2) of VRET for OCD aligns with patterns observed in anxiety disorder research, where VRET is comparable to *in vivo* exposure but does not consistently surpass *in vivo* exposure. In a randomized controlled trial (RCT) targeting contamination-related OCD, Javaherirenani et al. (54), found that the virtual reality exposure and response prevention (VRERP) group demonstrated significantly greater reductions in both obsession and compulsion scores on the Yale–Brown Obsessive–Compulsive Scale (Y-BOCS), with large effect sizes, as well as greater improvements in obsessive beliefs and functional disability compared to standard CBT. By contrast, Zadbar et al. (55), examined VR exposure therapy as a standalone intervention against a full course of therapist-delivered cognitive-behavioral therapy (CBT) incorporating ERP. While both groups exhibited statistically significant reductions in obsessive–compulsive symptoms, no significant between-group differences emerged, indicating that VR exposure was non-inferior, though not superior, to standard CBT/ERP in this context.

### Eating disorders

For eating disorders, VR appears to be a feasible adjunct that can match or exceed standard therapy on certain psychological outcomes, particularly body image and cue reactivity, however, the evidence base remains limited in size and scope (k = 4). In treatment-resistant bulimia nervosa and binge eating disorder (BED), Ferrer-García et al. (56), conducted an RCT comparing VR cue-exposure therapy with additional cognitive therapy sessions after non-response to initial CBT. The VR group demonstrated significantly greater reductions in binge frequency, food cravings, and global eating disorder psychopathology, as well as higher abstinence rates from binge eating at post-treatment, suggesting potential value as a second-line intervention. Earlier work by Riva et al. (57), evaluated Experiential Cognitive Therapy (ECT), a multifactorial program incorporating VR, against standard CBT and nutritional education groups in individuals with BED. At six-month follow-up, binge cessation rates were highest in the VR-based ECT group, compared to CBT and nutritional support alone. The ECT group also showed superior improvements on most psychometric measures, including eating disorder symptomatology (EDI-2) and body image scores.

In a trial targeting obesity with comorbid binge eating, Cesa et al. (58), evaluated an enhanced cognitive–behavioral therapy incorporating a VR protocol designed to “unlock” entrenched negative body memories in morbidly obese patients with BED, comparing it to standard CBT and an inpatient multimodal program. While all groups achieved substantial weight loss and binge-eating remission during the inpatient phase, only the VR-enhanced group maintained significantly greater weight loss at one-year follow-up. Both VR and standard CBT groups sustained low binge frequency over time, whereas the inpatient-only group showed greater relapse. Improvements in body satisfaction were also observed, with the VR-based approach outperforming CBT in preventing weight regain, though it did not confer superior control over binge eating. These findings suggest that VR interventions may offer particular value in enhancing long-term weight maintenance and addressing body image disturbances in BED.

### Substance Use disorders

Research examining the application of VR therapy in an RCT for addictions remains limited (k = 1), though preliminary evidence is promising, particularly in alcohol use disorder. The strongest support comes from randomized controlled trials employing VR-based cue exposure techniques. Zhang et al. (59), found that, compared to standard clinical treatment alone, VR-based cue exposure significantly reduced self-reported craving and heart rate in alcohol-dependent patients, though no effects were observed for skin conductance or respiration. These findings suggest short-term benefits for craving and certain physiological responses, with mixed effects across autonomic measures. These findings are consistent with earlier laboratory research demonstrating that VR can reliably elicit substance-related cravings, thereby providing an ecologically valid platform for exposure-based extinction of conditioned responses (60).

### Psychotic disorders (paranoia and social functioning)

With respect to psychotic disorders, VR interventions have been explored as adjunctive treatments. While these findings indicate that VR-CBT may offer a feasible and acceptable method for practicing exposure to feared social situations in a controlled and replicable environment, the evidence base remains limited (k = 2). In their RCT with VR-CBT with TAU and TAU alone, Pot-Kolder et al. (61), focused on graded exposures to social environments for individuals with psychotic disorders and recent paranoid ideation. While it did not significantly increase time spent with others, VR-CBT produced significant reductions in momentary paranoia (large effect) and anxiety (moderate effect) that were maintained at six-month follow-up. In a comparison between VR-CBT and TAU, Geraets et al. (62), found that VR-CBT led to greater reductions than standard care in daily-life paranoia (e.g., feeling suspicious, disliked, or hurt) and negative affect (e.g., feeling anxious, down, or insecure), though positive affect did not improve. These findings suggest that VR-CBT may help reduce certain symptoms of paranoia and anxiety in psychosis, though effects on broader social participation remain uncertain.

### Depressive and other disorders

While most VR research has focused on anxiety and related disorders, recent work has begun to explore its potential for depression, stress-related conditions, and sleep disorders. In a pilot randomized controlled trial, Paul et al. (63), conducted a small randomized controlled pilot trial comparing a VR-based behavioral activation program with both standard behavioral activation and treatment-as-usual for adults diagnosed with major depressive disorder. In the VR condition, participants used headsets to take part in enjoyable, goal-oriented activities within immersive virtual settings over four brief sessions. The VR approach was well-received, with high participation and no reported adverse effects, and it produced larger reductions in depressive symptoms (average PHQ-9 decrease of 5.67 points, moving from moderate to mild severity) than treatment-as-usual, with slightly greater gains than standard behavioral activation. While the modest sample size limits generalization, the findings indicate that VR-based activation may be a practical and engaging option for individuals who face challenges accessing or participating in real-world activities.

VR has also been applied to stress management across diagnostic categories. In a crossover trial, Veling et al. (64), tested a VR relaxation program (VRelax) against standard relaxation in psychiatric outpatients. Both reduced negative affect, but VR led to greater immediate decreases in anxiety and sadness, and larger boosts in cheerfulness. No differences were found for short-term stress or symptom changes, indicating VR's advantages may be limited to momentary mood improvements. Preliminary evidence also supports VR use in sleep disorders. Wan et al. (65), tested a six-week VR intervention against a control condition in individuals with chronic insomnia. The VR group demonstrated greater improvements in sleep quality and insomnia severity, as well as reductions in comorbid anxiety and depressive symptoms. While encouraging, these findings require replication in larger, more diverse samples to assess durability and generalizability.

## Discussion

The present systematic review provides a comprehensive evaluation of VR therapies across a variety of mental health disorders, addressing several key limitations of previous reviews. Earlier analyses often included heterogeneous study designs and relied on both clinical and subclinical samples. In contrast, this review was restricted exclusively to RCTs, the gold standard for establishing treatment efficacy (66), and to studies employing standardized diagnostic criteria, thereby enhancing methodological rigor. By consolidating evidence from 41 RCTs encompassing anxiety, mood, trauma-related, obsessive-compulsive, eating, substance use, and psychotic disorders, this review extends the evidence base well beyond its traditional applications in phobia and anxiety disorders. Overall, the results indicate that VR-based therapy is superior to no treatment, control conditions, or waitlist groups, supporting VRs clinical utility across diverse diagnostic categories. However, when compared with established evidence-based interventions, particularly CBT and exposure-based therapies, the findings remain mixed, suggesting that while VR represents a promising and efficacious adjunct treatment, its comparative advantage and long-term generalizability remain to be determined.

Considering specific phobias, where VRET has been most applied, results have found VRET to be as efficacious as *in vivo* exposure, similar to previous reviews regarding acrophobia, fear of flying, spider or small animal phobias (15–17). However, the current review also failed to establish whether VRET is superior to existing standardized evidence-based treatments for specific phobias. One study (36) established long term gains of VRET compared to *in vivo* exposure in terms of test-flights and at follow-up assessments while the gains plateaued in the imaginal exposure group at follow-up. This may indicate a modest advantage of VR in consolidating real-world confidence. Overall, the evidence supports VR exposure as a safe and effective intervention for specific phobias, matching the efficacy of *in vivo* exposure while outperforming inactive controls.

With respect to social anxiety disorder, overall, the evidence indicated that VR exposure therapy was an effective modality, with efficacy generally comparable to *in vivo* exposure. Variability in effect sizes across studies appeared to be driven less by the fundamental capacity of VR to reduce social anxiety, and more by factors such as therapist involvement, the realism and interactivity of virtual scenarios, and whether VR is delivered in isolation or as part of a broader cognitive-behavioral framework. Some results (48) indicated that VR can match, and in certain aspects potentially enhance, the benefits of traditional exposure when integrated into pharmacological and psychological treatment. Similarly in terms of panic disorder with agoraphobia, VR exposure yields clinically meaningful symptom reduction and, in most studies (17, 49) matches the efficacy of gold-standard *in vivo* methods. Variability in outcomes, ranging from strict equivalence to a modest advantage for *in vivo* exposure, likely reflect methodological differences, sample sizes, and the degree of technological sophistication used in VR delivery. Importantly, no evidence suggests that VR is inferior to *in vivo* exposure; at minimum, it offers a viable alternative with added advantages such as precise control of stimuli and greater acceptability for patients reluctant to engage in real-world exposures at treatment outset.

Apart from anxiety disorders, the current evidence base supports VR exposure as a viable alternative delivery format for exposure therapy in PTSD. Across head-to-head trials, VR typically performs comparably to imaginal exposure, with inconsistent evidence for any incremental benefit (50, 51). Indications of advantage appear most plausible in specific contexts (e.g., patients struggling to engage with imaginal methods or treatment-resistant presentations), but these findings come from designs and comparators that limit generalizability (52, 53). Acceptability and safety profiles in VR trials generally resembled those of standard exposure, with low reported rates of adverse events and infrequent cybersickness under modern protocols; however, small sample sizes and heterogeneous designs limit precision in estimating rare adverse effects. Patient preferences were also heterogeneous, with some reporting that VR's multi-sensory cues made it easier to engage, while others did not perceive a clear difference compared to imaginal work (50–53). Given the heterogeneity in samples, protocols, and controls, conclusions about differential efficacy should be considered provisional pending larger, methodologically comparable trials.

Current evidence also supports VR as a feasible and efficacious modality for both eliciting and diminishing OCD-related distress, particularly for simulating contamination scenarios that may be impractical or unacceptable *in vivo*. However, the absence of consistent superiority over conventional ERP strongly emphasizes the need for methodologically rigorous trials, especially targeting under-researched OCD dimensions such as checking, hoarding, or intrusive taboo thoughts. Across studies related to eating disorders, VR exposure tasks, such as interacting with feared foods in a simulated kitchen or viewing one's virtual avatar, appeared to elicit relevant affective and cognitive responses (e.g., anxiety, urge to binge or restrict) in a controlled context, enabling targeted cognitive restructuring and coping practice. Importantly, while several trials report VR-related advantages over control conditions, some outcomes (e.g., weight loss in (58) showed no incremental benefit. Follow-up durations also varied from short-term to 12 months, with generally positive but not uniformly superior maintenance effects. VR appears to be a feasible adjunct that can match or exceed standard therapy on certain psychological outcomes, particularly body image and cue reactivity, but further research across diverse eating disorder presentations and with rigorous long-term follow-up is needed to substantiate its efficacy.

For substance use disorders, not all studies found VR superior to standardized interventions. Long-term clinical outcomes, such as sustained abstinence, remain insufficiently examined, and the generalizability of short-term craving reductions to real-world relapse prevention is yet to be established. Overall, while the current evidence base is preliminary, findings suggest that VR cue exposure represents a feasible and acceptable adjunct to standard addiction treatment, with potential utility in addressing cue-triggered cravings in a controlled and immersive context. Similarly, for psychotic disorders, VR-CBT may offer a feasible and acceptable method for practicing exposure to feared social situations in a controlled and replicable environment, the evidence base remains limited. The existing trials come primarily from a single research group, with relatively small samples. Long-term impacts on broader functional outcomes require further study. Emerging evidence suggests that VR may offer benefits for depression, stress reduction, and insomnia through mechanisms such as enhanced engagement, immersive relaxation, and structured behavioral practice. Nonetheless, sample size limitations, variable outcome durations, and the need for direct comparisons with established treatments underscore the importance of cautious interpretation.

The use of VR in treatment has multiple practical implications for mental health care in general. For instance, Hidayat et al. (27) discuss how VR overcomes limitations of conventional therapy by offering potential solutions for realistic exposure. Accessibility and cost-effectiveness of VR also make it an appealing treatment modality (26). VR is also a testament to capture the reduction in treatment gap with the use of technology as outlined across the articles in terms of its effectiveness being similar to traditional talk therapy. In consideration of all the above mentioned aspects, this systematic review makes adequate grounds for the need to build standardization across studies using VR as a therapeutic intervention for psychological disorders. Across all diagnoses, the lack of consistency of VR as a therapeutic tool raises concerns regarding the real-world application of VR. The lack of consistency across studies, especially in terms of the VR paradigms, makes it difficult to draw substantial conclusions and generalizability regarding VR based therapy as a successful therapeutic intervention.

Almost every study uses a different VR mechanism across software and hardware. The volume of programs available for VR modalities makes it difficult to ascertain which program is efficacious than the other. For example, in one RCT, the VR mechanism was immersive with elements of tactile augmentation (37), while in another, there was a combination of computer and smartphone interface for administration of the VR (67). In terms of hardware, while most used head-mounted displays, some also used computer displays, tactile elements, and biofeedback elements. Similarly, with software, some studies used pre-existing VR environments like the Virtual Reality for Eating Disorder Modification (57), NEUROVR (58), Delft Remote Virtual Reality Exposure Therapy (44), whereas some studies also created virtual environments for administration. The description of the hardware and software in the studies also lacked consistency with some studies not describing or elaborating on the software and hardware used.

The use of VR further lacks standardization in terms of session length, number of sessions, and total duration treatment, even within the same psychological disorder. In some studies, the number of VR sessions were fixed (48, 53, 62) while in other studies, a range was provided (37, 68). Some studies also stated that a few sessions from a larger number of sessions were VR (47, 69). This inconsistency makes it difficult to draw conclusions about the number of sessions that would be needed for treatment using VR. The total number of sessions using VR across the studies ranged from 1 to 16. In addition to the number of sessions, the duration of sessions also varied drastically across the studies, ranging from 8 min (55, 59) to 3 h (39). Many studies did not mention the duration, and in most studies with longer duration there was no clear indication of how much time was spent on VR as compared to other aspects.

Another important aspect, mainly focusing on the content of the VR, not clearly elucidated in some studies, is the use of VR as a tool in therapy vs. its use as a therapeutic intervention in itself. As this review focuses on VR as a therapy, its integration with standardized evidence-based interventions was of utmost importance. Exposure therapy was integrated in most studies, with some studies including CBT. However, the use of VR varied in terms of being used throughout the administration of the evidence-based therapy. For example, in one study, VR was used to practice skills like decision making and problem solving in relation to cue-exposure therapy, but this was in addition to the standard CBT (58). Another study also had a virtual therapist as a part of the VR providing instructions, coaching and repeated prompts (68). However, across the studies, there is no standardization regarding the evidence-based modalities used as well as the specific content delivered using VR. To determine the efficacy of VR in the treatment of psychological disorders, establishing standard protocols for number of sessions, duration of VR use, content delivered using VR, and the integration of VR in a systematic manner is essential.

The gap with respect to standardized diagnosis of psychological disorders is addressed adequately in this review, however, a gap in terms of the use of standardized outcome measures was further discovered. This makes the comparison across studies, calculating effect sizes for a large sample meta-analysis, and generalizability of results extremely difficult. As a next step, the authors of this review propose establishing strong guidelines for the trials aiming to establish the efficacy of VR for all psychological disorders. This would overcome the limitation of this paper by allowing calculations of mechanism of change in terms of effect sizes as a meta-analysis.

Taken together, the current evidence base, though promising, remains limited in size and scope. Additionally, the need for specialized equipment and trained facilitators may constrain scalability. Further multi-site, adequately powered trials are needed to establish the robustness and generalizability of these promising effects across diverse patient populations and clinical settings. With the shift in the world towards digital therapeutics, addressing the gaps in literature, enhancing the rigor and standardization in the RCTs, and establishing strong generalizable results would form a compelling base of evidence.

## Data Availability

The original contributions presented in the study are included in the article/Supplementary Material, further inquiries can be directed to the corresponding author.
